# Updating impairments and the failure to explore new hypotheses following right brain damage

**DOI:** 10.1007/s00221-018-5259-6

**Published:** 2018-04-12

**Authors:** Elisabeth Stöttinger, Carolyn Louise Guay, James Danckert, Britt Anderson

**Affiliations:** 1 Department of Psychology, Center for Cognitive Neuroscience, University of Salzburg, Hellbrunnerstrasse 34, 5020 Salzburg, Austria; 2 Department of Psychology, University of Waterloo, Waterloo, N2L 3G1 Canada; 3 Centre for Theoretical Neuroscience, University of Waterloo, Waterloo, N2L 3G1 Canada

**Keywords:** Right brain damage, Mental representations, Updating failures, Picture morphing, Exploration

## Abstract

We have shown recently that damage to the right hemisphere impairs the ability to update mental models when evidence suggests an old model is no longer appropriate. We argue that this deficit is generic in the sense that it crosses multiple cognitive and perceptual domains. Here, we examined the nature of this updating impairment to determine more precisely the underlying mechanisms. We had right (RBD, *N* = 12) and left brain damaged (LBD, *N* = 10) patients perform versions of our picture-morphing task in which pictures gradually morph from one object (e.g., shark) to another (e.g., plane). Performance was contrasted against two groups of healthy older controls, one matched on age (HCO-age-matched, *N* = 9) and another matched on general level of cognitive ability (HCO-cognitively-matched, *N* = 9). We replicated our earlier findings showing that RBD patients took longer than LBD patients and HCOs to report seeing the second object in a sequence of morphing images. The groups did not differ when exposed to a morphing sequence a second time, or when responding to ambiguous images outside the morphing context. This indicates that RBD patients have little difficulty alternating between known representations or labeling ambiguous images. Instead, the difficulty lies in generating alternate hypotheses for ambiguous information. Lesion overlay analyses, although speculative given the sample size, are consistent with our fMRI work in healthy individuals in implicating the anterior insular cortex as critical for updating mental models.

## Introduction

Damage to the right hemisphere results in a range of heterogeneous impairments, including impoverished spatial attention (i.e., neglect; Danckert et al. 2012a), statistical learning (Shaqiri and Anderson 2013; Shaqiri et al. 2013), humor appreciation (Brownell et al. 1983), working memory capacity (Ferber and Danckert 2006; Husain et al. 2001), and deficient Theory of Mind (Happé et al. 1999; Griffin et al. 2006; Weed et al. 2010). We have argued that these impairments can be parsimoniously explained by a general impairment in updating mental models (Danckert et al. 2012a, b; Filipowicz et al. 2016; Geng and Vossel 2013; Shaqiri and Anderson 2013; Shaqiri et al. 2013; Stöttinger et al. 2014; Vocat et al. 2012).

Everyday we are confronted with an enormous amount of information. Mental models allow us to deal with this complexity by compactly representing the regularities that govern our environment. We use these models to guide our decisions (e.g., Is the situation dangerous? Is this food edible? Johnson-Laird 2004; Griffiths and Tenenbaum 2012; Tenenbaum et al. 2011). Since the world is in flux, these models are only useful if we update them when things change. While a salient shift in the environment allows us to simply react (e.g., a bolt of lightning indicates that it is time to head indoors), gradual changes in the environment require the aggregation of mismatching evidence to provoke a proactive decision (e.g., when does cloud cover become sufficiently menacing to indicate a coming storm? McGuire et al. 2014).

We previously showed that right brain damage resulted in selective impairment of gradual updating in response to small, subtle environmental changes. We had left (LBD) and right brain damaged (RBD) patients play the children’s game of ‘rock, paper, scissors’ (RPS) against a computer opponent. The computer initially played randomly before switching to ‘paper’ 80% of the time. While LBD patients and controls rapidly adopted the optimal strategy (i.e., scissors), RBD patients failed to respond to the transition, with most patients continuing to play randomly (Danckert et al. 2012b). Similarly, RBD patients showed a severe updating impairment in a gradually morphing picture task, in which one object (e.g., shark) morphed over several iterations into a completely different object (e.g., plane; Fig. 2, top panel). The logic was to first provide participants with a mental model (e.g., “It’s a shark”) and then evaluate how much evidence was required for them to update to a new mental model (e.g., “It’s a plane now”). RBD patients needed significantly more evidence (i.e., more pictures) before they reported seeing the second object compared to LBD patients and healthy controls. Performance in the RPS task correlated with performance in the picture morphing task, indicating a general updating impairment across multiple cognitive domains (Stöttinger et al. 2014).

While our prior studies showed an updating impairment after RBD, they did not unequivocally demonstrate the mechanism for this impairment. Updating to gradually evolving stimuli requires several different processes: (1) we need to be able to detect and accumulate evidence of a mismatch. (2) When the mismatch reaches some threshold we need to explore for potential alternative interpretations. (3) Finally, we need to proactively switch to this new interpretation. The aim of the research reported here was to test for the integrity of each of these mechanisms in RBD patients.

From our prior study, we know that RBD patients notice small changes in the sequences of morphing images. RBD patients, however, incorporated changes into their initial perceptual representation (e.g., “The shark’s fins are getting bigger.”), rather than entertaining an entirely novel interpretation (e.g., fins have now become wings). Similarly, within the context of the rock, paper, scissors game, although RBD patients fail to update to a change in strategy, their own play choices do reflect the fact that they have noticed something has changed. That is, RBD patients rapidly abandon their old play strategy but fail to adopt a new strategy optimized for the change in their opponent’s bias (Stöttinger et al. 2014). In combination, these data suggest that the impairment in updating following RBD may be due to either (1) a failure to explore alternative interpretations in light of observed changes or (2) a general inability to proactively switch.

To evaluate these hypotheses, RBD and LBD patients as well as healthy seniors (HCO) were presented with three versions of our picture morphing task. The hypotheses and predictions for this study are summarized in Table 1. In the gradual condition participants saw four picture sets in which one object (e.g., shark) morphed into a different object (e.g., plane; replication of Stöttinger et al. 2014; Fig. 2, top row). In the repeat condition, some of these sequences were repeated in the reverse order (e.g., plane-to-shark). In the random condition, images of four additional morphing sequences were presented in random order with all pictures intermixed. For example, a participant would see the second picture of the gun-hair dryer picture set, followed by the ninth picture of the spider-sun picture set, and so on (Fig. 2, middle row). We compared the likelihood that participants reported the first or second object for pictures based on their order in the morphing sequence (i.e., 1st to 15th picture position), and on the type of sequence (i.e., gradual, repeat or random).

**Table 1:** Expected outcome vs. actual outcome

	Exploration	Proactive switch	Perceptual/attentional bias
Test (condition)	ΔRandom–gradual	Repeat	Random
Expected	RBD < LBD&HCO	RBD > LBD&HCO	RBD **≠** LBD & HCO
Actual	RBD < LBD&HCO ✓	RBD = LBD&HCO ✗ (?)	RBD = LBD & HCO ✗

Summary of expected vs. actual results for all three hypotheses (i.e., exploration, proactive switch, perceptual/attentional bias) potentially explaining the updating impairment seen in RBD patients. The second row refers to the condition that was used to evaluate the hypothesis. Please note that results are only displayed for HCO matched on cognitive status

Each of our key hypotheses was addressed in the following ways:

1. To evaluate whether updating impairments seen in RBD patients can be explained by a failure to explore, we calculated the difference in performance between the random–gradual conditions. We know that healthy individuals tend to switch categories earlier in the morph series when presented in a gradual context (e.g., when the object is still 60% shark and only 40% plane) than when single images from the same sequence are presented in isolation, outside the morphing context (Egré et al. 2013; Raffman 2011; Stöttinger et al. 2016; see Egré et al. 2018 for review). This suggests that healthy individuals are exploring alternative interpretations conditional on the evolving history they have viewed (e.g., “I know it was a shark but what else could it be? A bird?”). In the context of the rock–paper–scissors game, we found that RBD patients explored a limited selection of alternative strategies (Danckert et al. 2012b) and employed less efficient exploration strategies (Sepahvand et al. 2014). If updating impairments in RBD patients are due to a generic failure to explore alternatives evident across different tasks and domains, we expect them to show no benefit from the contextual information provided by the gradual morphing sequence (Egré et al. 2013; Raffman 2011; Stöttinger et al. 2016; Table 1, second row).
2. To evaluate whether RBD patients would always struggle to *proactively shift* to a new interpretation, (irrespective of whether they are naïve as to the second object), we compared performance in the repeat condition across participant groups. Evidence that RBD impairs the ability to make proactive decisions comes from research using ambiguous or bistable figures such as the Rubin’s face/vase picture or the Necker cube in which healthy individuals typically alternate between two mutually exclusive interpretations (see Long and Toppino 2004 for review). Given that the sensory input is stable, the switch to a new interpretation is internally generated by the participant. Research shows that performance in an ambiguous figures task of this kind is associated with the right hemisphere, with damage to right frontal cortex resulting in significant impairment in switching to the second interpretation (Meenan and Miller 1994). Furthermore, right frontal and parietal areas are active during proactive alternations between two percepts (Britz et al. 2009, 2011; Sterzer and Kleinschmidt 2007; Weilnhammer et al. 2013; Zaretskaya et al. 2010). So if RBD patients have a general impairment in proactively switching to a new interpretation irrespective of whether or not they know what the second object will be, we expect them to identify the second object later compared to other participant groups in the repeat condition (Table 1, third row).
  At this point, it is also worth noting that gradual updating should not be confused with set-shifting. While participants have to react to a salient *mismatch* in the Wisconsin Card Sorting Test (WCST; i.e., an action that was considered “correct” suddenly becomes “incorrect”; Grant and Berg 1948), updating in our tasks rests on the accumulation of gradual changes in a noisy environment; participants proactively decide at which point their current model is no longer supported by the evidence. Indeed, we found that while RBD patients showed a selective updating impairment, there was no difference between RBD and LBD patients in a card sorting test akin to the WCST (Danckert et al. 2012b; Stöttinger et al. 2014; Piper et al. 2012).
3. Lastly, to assess the contribution of general post-stroke visual or attentional impairments (Hepworth et al. 2015), we compared the performance of RBD patients with the performance of control groups in the random condition. Damage to the right hemisphere often results in an attentional bias towards the ipsilesional side (i.e., neglect; Danckert et al. 2012a). This could have potentially hindered the capacity of RBD patients to correctly identify small changes in our original study, and consequently to update to a new model. If the updating impairment in RBD patients is due to a general perceptual and/or attentional impairment, we expect RBD patients to perform differently than LBD patients when pictures are presented individually outside of the morphing context (i.e., random condition; Table 1, right column).

One major assumption of our study is that the benefit for the gradual over the random presentation sequence is indicative of active exploration. While this benefit has also been reported elsewhere in the literature (see Egré et al. 2018 for review), to our knowledge our study was the only one that showed this benefit in the context of morphing objects (Stöttinger et al. 2016). In the original study, we used a between subjects design. We therefore first evaluated in an online study whether we can replicate the effect when the type of presentation (gradual vs. random) is manipulated within participants—a non-trivial point given the ongoing discussion about failures to replicate in psychological science (Bohannon 2015).

## Methods

Prior to the patient study, we conducted an online study to determine the benefit of gradual presentations in a within subject design (Stöttinger et al. 2016). The same picture sets used in the online study were then used in the patient study. Besides the need to replicate the effect, the online study also allowed us to have a reference point for the performance of healthy, younger individuals. Results of the online study are included in the graphs depicting the results of patients and healthy seniors for comparison purposes.

### Participants

#### Online study

Seventy-seven participants (33 female) recruited through Mechanical Turk, and between 19 and 53 years of age (mean 35.34, SD 9.62) participated in this study [Caucasian/white (80.50%), Hispanic (7.8%), African American (5.2%), East Asian (3.9%)]. Participants received $1.50 for their participation.

#### Patient study

**Fig. 1:**
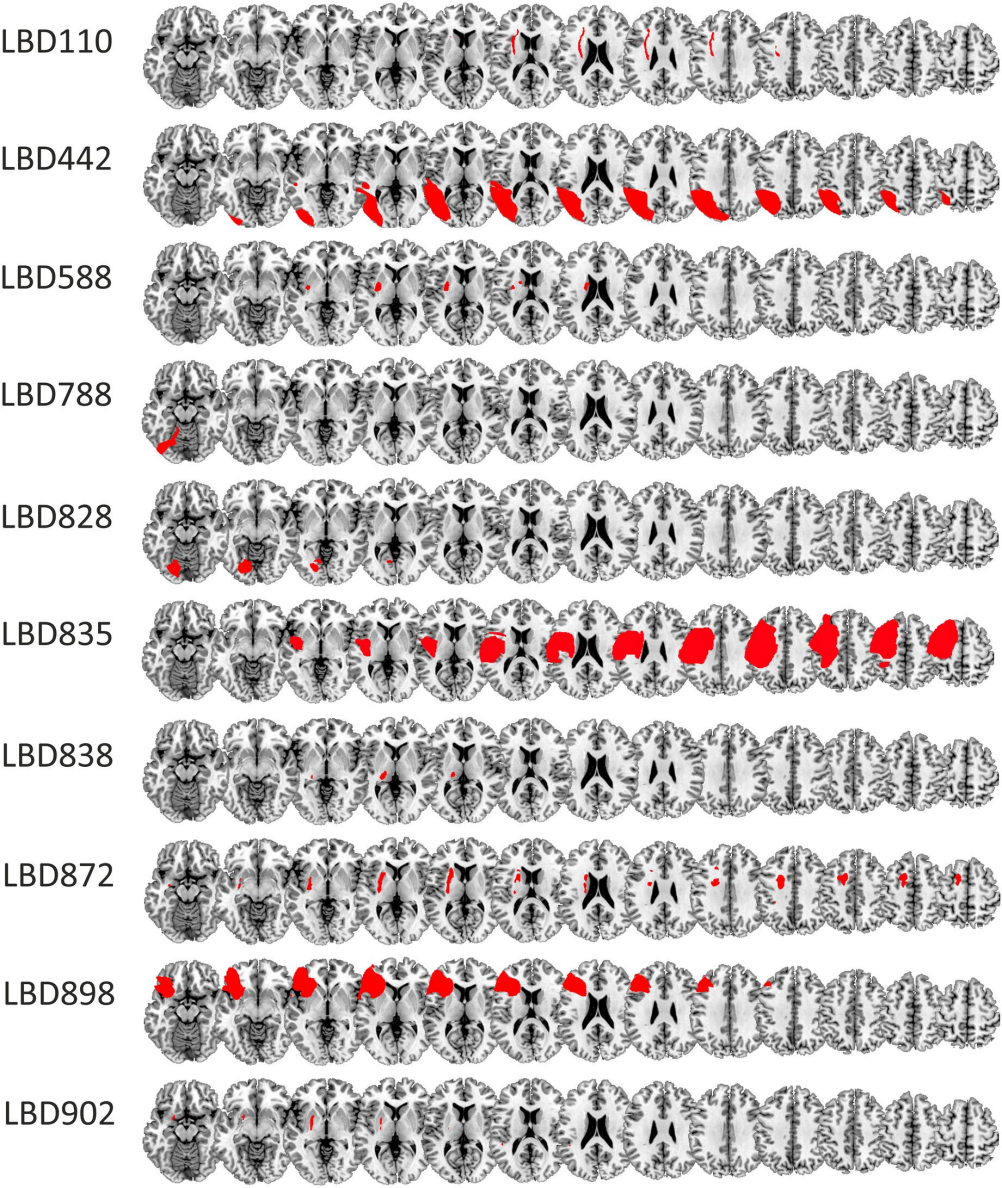

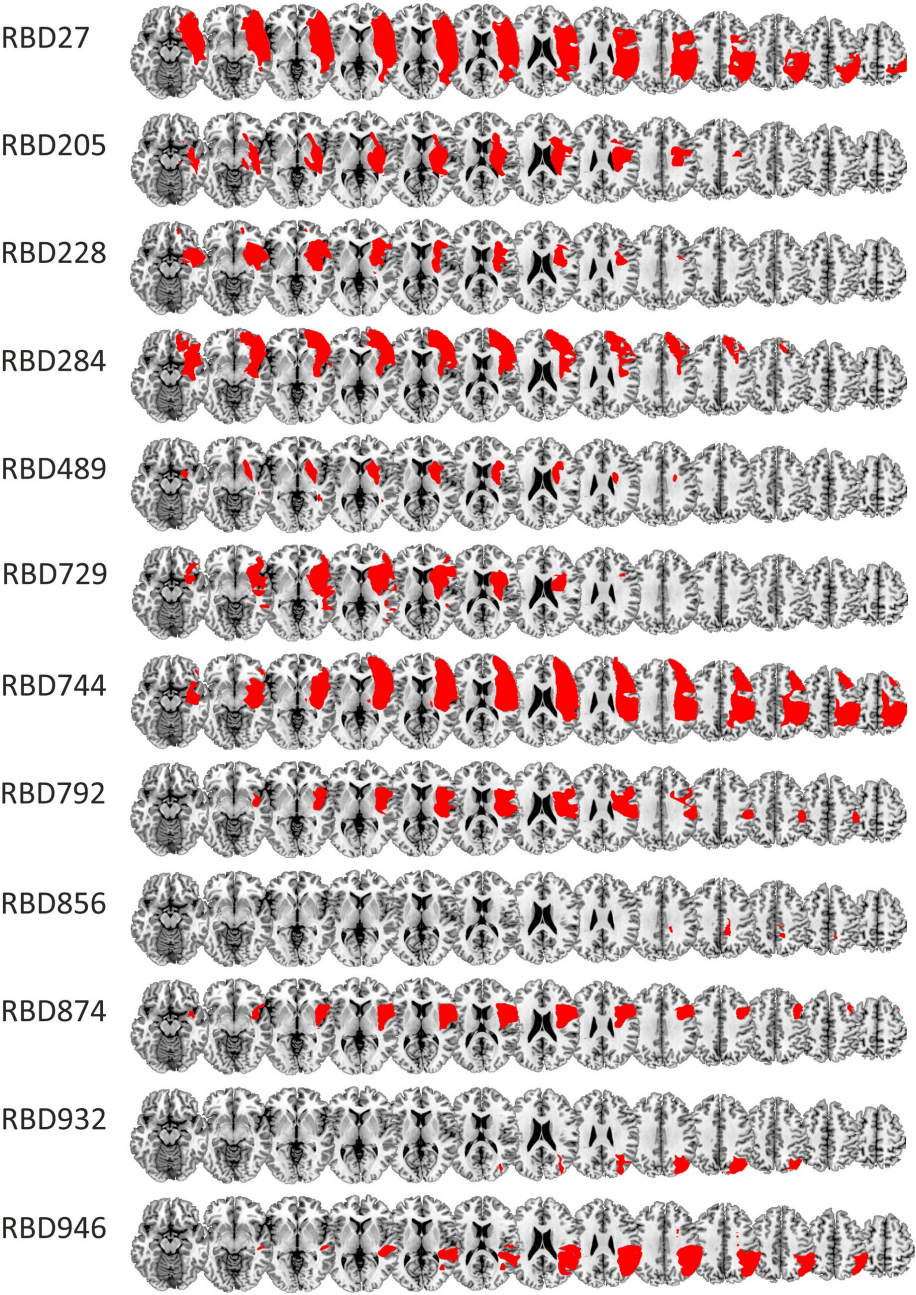
Lesion tracings RBD (**a**) and LBD patients (**b**) superimposed on the MNI template. Lesions shown in neurological convention

Four groups were tested in this study—RBD patients, LBD patients, younger (≤ 70 years; i.e., HCO-age-matched) and older (> 70 years; i.e., HCO-cognitively-matched) healthy seniors. Patients were recruited from the Neurological Patient Database of the University of Waterloo (Heart and Stroke Foundation funded). Of the 13 LBD patients three were excluded due to Montreal Cognitive Assessment (MoCA; Nasreddine et al. 2005) scores in the demented range (*N* = 2; MoCA ≤ 9). This score was considerably lower than the optimal cutoff point for vascular dementia (i.e., MoCA < 17; Freitas et al. 2012). One patient was excluded due to a failure to find any discernible lesion on available brain scans (*N* = 1). The final sample of LBD patients comprised ten patients (2 female, mean age 61.28 years, ± 14.47). Nine of these patients were stroke patients. In one patient (#835), brain damage was due to a resection of an arterial vascular malformation. Twelve RBD patients (2 female, mean age 65.88 years ± 10.13) participated. All of them were stroke survivors. Demographics for all patients are in Table 2 with lesions shown in Fig. 1. Patients were screened for neglect upon admission to the database and again prior to the experiment using the Behavioral Inattention Test (BIT; Wilson et al. 1987). Six RBD patients showed neglect at initial screening, with two showing chronic neglect at testing (#284, #744). No LBD patient showed neglect. Six of the ten LBD patients were reported to have aphasia when admitted to the database. One LBD patient experienced slight word finding difficulties at testing. He was able to describe all the objects and his answers could be reliably coded as either the first or the second object.

**Table 2:** Demographics for patients (a) and HCO (b)

ID	Lesion volume	Age (years)	Time since stroke (years)	MoCA	Gender	Education (years)
(a) Demographics (patients)						
LBD patients						
110	65	64	8.67	23	Male	12
442	8127	68	12.44	23	Male	14
588	274	68	3.25	28	Male	20
788	328	35	1.84	22	Male	15
828	7374	76	1.44	25	Female	17
835	7573	35	1.39	25	Male	14
838	105	69	1.41	26	Male	23
872	665	71	0.89	23	Male	10
898+	5396	66	0.48	23	Male	8
902+	161	61	1.76	29	Female	12
RBD patients						
27^a^	21,187	49	8.37	27	Male	15
205^a^	3742	62	6.69	29	Male	14
228	6316	86	6.62	26	Female	11
284^b^	14,203	74	6.95	22	Female	12
489^a^	1693	71	4.36	24	Male	15
729^a^	5758	66	2.30	22	Male	14
744^b^	21,479	73	3.60	25	Male	24
792	5878	64	1.67	24	Male	15
856	232	55	0.87	26	Male	16
874	5026	56	1.16	26	Male	14
932	1520	62	0.26	25	Male	16
946	5694	72	0.20	18	Male	20

ID	Age (years)	MoCA	Gender	Education (years)
(b) Demographics (healthy controls)				
Younger healthy controls (≤ 70 years)				
1	61	28	Female	20
46	64	28	Female	17
110	69	27	Female	17
143	68	30	Female	16
193	62	30	Female	12
230	69	28	Female	18
249	70	25	Female	24
351	67	25	Female	16
408	68	29	Female	16
Older healthy controls (> 70 years)				
3	76	25	Male	17
32	76	22	Female	13
37	76	27	Female	17
148	81	23	Female	22
206	80	26	Male	20
208	81	28	Female	14
321	81	30	Female	18
369	83	25	Female	13
409	73	26	Male	16

^a^Neglect at time of screening (^b^and at time of testing)

Nineteen healthy older controls were recruited from the University of Waterloo’s Research in Aging Participant Pool. This pool recruits community dwelling seniors for participation in studies on aging. Thus, our HCO were enrolled based on age. We subdivided our healthy older controls into two subgroups. One matched to our clinical participants based on age, and another based on MOCA scores to equate cognitive ability. The former group (HCO-age-matched) was under 70 years of age, while the second subgroup (HCO-cognitively-matched) was over 70 years of age. After recruitment one of the healthy elders was found to have had a past neurological illness, and was excluded from further analyses. The final sample of 18 participants consisted of nine who were 70 years or younger (HCO-age-matched: 9 females, mean age 66.35 years, ± 3.35) and nine older than 70 years (HCO-cognitively-matched: 6 female, mean age 78.55 years, ± 3.32). The HCO-cognitively-matched control group had a nominally lower MOCA score than the HCO-age-matched group, but the two control groups did not differ statistically {HCO-cognitively-matched mean = 25.78, ± 2.44 vs. HCO-age-matched mean = 27.78, ± 1.86; [*t*(16) = 1.96, *p* = .068]}. Six of the HCO had a MoCA score between 25 and 22, potentially indicating a mild cognitive impairment (Smith et al. 2007; Nasreddine et al. 2005; but see; Lee et al. 2008 and; Luis et al. 2009 for the argument of lower cut-off points).

The MoCA for LBD (mean 24.79, ± 2.36) and RBD patients (mean 24.50, ± 2.84) was lower than that of the HCO-age-matched group (all *p*’s < .01), but comparable to that of the HCO-cognitively-matched group (all *p*’s > .25). There was no significant difference in MoCA scores of LBD and RBD patients [*t*(20) = 0.18, *p* = .86]. LBD and RBD were of comparable age [*t*(20) = 0.87, *p* = .40]. Both groups were significantly younger than the HCO-cognitively-matched group (all *p*’s > .01) but comparable in age to the HCO-age-matched group (all *p*’s > .30). Also, there was no significant difference for time since stroke or lesion volume between the RBD and LBD patients (all *p*’s > .05).

The University of Waterloo’s Office of Research Ethics approved the protocol for both studies—online and patient study. Participants on both studies gave informed written consent prior to participation according to the Declaration of Helsinki, by either clicking on the “I agree” button (online study), or by signing the consent letter (patient study).

### Stimuli and design

Each participant saw eight picture sets selected from a larger set validated in an earlier study (Stöttinger et al. 2016; https://osf.io/qi2vg/). All images were a standard size (316 × 316 pixels) displayed on a white background. Four sets were presented in a gradual order, four in random order. In the gradual condition one common object morphed over 15 iterations into a different object (Stöttinger et al. 2014, 2016). Two of these sets were presented again, but in reverse order (i.e., repeat condition).

**Fig. 2:**
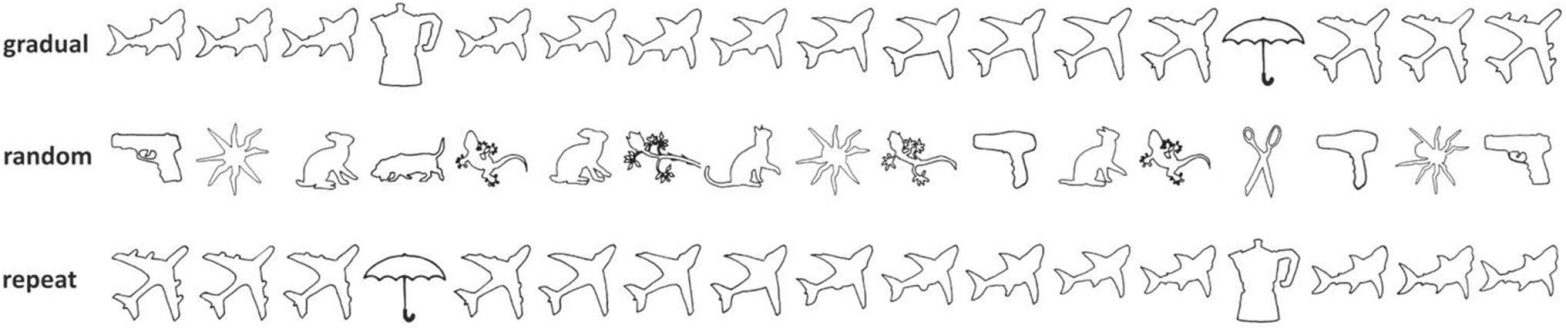
Objects morphed over 15 iterations from object 1 into object 2 (gradual condition), or vice versa (repeat condition). In the random condition, objects were presented individually, outside of the morphing context (e.g., second picture of the gun-hair dryer picture set the ninth picture of the spider-sun picture set, etc.)

In the random condition, four picture sets were presented in a random order. Pictures from these sets were randomly assigned to four new series with 15 pictures in each. Each random series contained the same number of pictures from each picture set and each part within a series. That is, each random series had five pictures from the first third of the morphing series (pictures #1 to #5); five pictures from the middle (pictures #6 to #10), and five pictures from the end of each series (pictures #11 to #15). In each of the eight picture sets, one additional object was presented after the third and twelfth pictures as catch trials. Catch trials assessed whether participants were simply perseverating (Fig. 2; Stöttinger et al. 2016).

The presentation of each set (gradual or random), the order of presentation (random then gradual or vice versa), as well as order of the series (series 1, 2, 3, 4, 1, 3 vs. 3, 1, 4, 2, 1, 3), was varied between participants resulting in eight versions. Versions were counterbalanced across participants.

### Procedure

#### Online study

The online study questionnaires were designed using Qualtrics©. Participants first filled out demographic questions before being assigned to one of eight task versions using the randomize function of Qualtrics©, and yielding roughly the same number of participants for each task order (*n* = 39 did random, gradual, repeat and 38 did gradual, repeat, random). Prior to the gradual condition, participants were informed they would see six different picture series, containing 17 images and that each series would begin with a commonly known object before changing gradually to show a different object by the end of the series. In the random condition, participants were told they would see 68 pictures of objects. In all conditions, participants saw one picture at a time and typed their answer underneath the picture. The next picture was revealed by clicking a button at the bottom of the screen. On average, participants needed 20 min (± 15 min) to complete the questionnaire. Time to complete the questionnaire did not correlate with any of the dependent measures in our task (all *p*’s > .05).

There were a small number of technical glitches (< 1% of all image presentations). For 29 image presentations (0.22%), the wrong picture was presented at the first picture position of a series (e.g., a saw instead of a shark). From our initial study, we know that these pictures are never rated as the “second object”. These items were therefore coded as “first object” reports. In one version, (a picture of a saw was presented instead of the correct picture at picture position #10). If this happened between a switch of percepts, the set was removed (seven individual cases). Otherwise, it was rated as the answer which preceded and followed the erroneous presentation. Infrequently, participants failed to answer (i.e., 0.19% of images). Most of these omissions were preceded and followed by the same response, suggesting no change in the participant’s conscious percept. In ten cases, the omission occurred between a switch in report. These sets were removed from further analysis. Importantly, performance in the current online study was the same as in Stöttinger et al. (2016) (all *p*’s > .05).

#### Patient study

All assessments and tasks were done in the same order for each patient: BIT, MoCA, picture morphing task. Participants were tested individually in a room at the University of Waterloo. In a few cases, participants were tested at their own home. Instructions were presented on a screen and repeated verbally to the participants before each condition in the picture morphing task. Participants saw one picture at a time and were asked to tell us for each picture what they saw. Answers were recorded and transcribed after the experiment. Each picture was preceded and followed by a fixation cross. Patients and healthy participants received $10 per hour of study for their participation.

### Data analysis

#### Behavioral data

Responses were coded as seeing the “first object”, or seeing an object other than the first object (i.e., “second object”). In addition, we evaluated whether the catch trials were identified correctly. For repeated sequences, the coding as first or second object was done based on the ordering used in the morphing sequence. For example, when a picture set morphed from a shark to a plane in the gradual condition, and from a plane to a shark in the repeat condition, “shark” was coded as first object in the gradual condition, with “plane” coded as first object in the repeat condition. While the coding was not done blindly, it did use a list of validated picture terms from Stöttinger et al. (2016; https://osf.io/qi2vg/), and was done independently by the first two authors with an interrater agreement of 98.98% for the online study and 99.13% for the patient study. The high agreement is explained by the fact that there was little opportunity for ambiguity. The majority of answers in the online study were less than three words (one word, e.g., “frog”; 90.45%; two words, e.g., “Jumping man”; 8.81%; or three words, e.g., “man jumping up”; 0.47%). In only 0.28%, the answer included more than three words. Most answers in the patient study were also less than four words (73%). Participants in the patient study, however, were more prone to longer answers with 9.44% containing ten words or more.

On a few occasions (2.18%), patient responses indicated more than one object (e.g., “jet turning into a shark”). Most of these were in the random condition (only 0.63% of individual responses in the gradual or repeat condition). Following the procedure of our initial study (Stöttinger et al. 2014), answers in the gradual and repeat condition were coded as “second object” as soon as the second object was mentioned. In 1.54% of individual responses both objects were named in the random condition. In cases where the participant stated explicitly which object he/she preferred (e.g., “It could be a cat or a rabbit. But it looks more like a rabbit”) answers were coded based on the indicated preference. Applying this rule, 0.51% of individual answers were coded as “first object”, and 0.44% of individual answers were coded as “second object”. When answers could not unambiguously assigned to either object (e.g., “airplane or shark possibly”), answers were always coded as “second object” (0.32%). We repeated analyses in Sect. Difference: random minus gradual using only the coding of when patient participants first reported the second object (and ignoring a stated preference) with no change in the pattern of results or statistically significant findings.

#### Lesion tracing

The most recent available clinical CT (17) or MRI (five) scan was obtained for each patient. All scans were aligned to the anterior commissure in SPM8. Lesions were traced manually in MRIcron (Rorden et al. 2007) and spatially normalized using the Clinical Toolbox in SPM (Rorden et al. 2012). Common involvement of brain-damaged regions across different patient groups was identified by overlapping individual normalized brain lesions on a standard template (i.e., AICHA—An atlas of intrinsic connectivity of homotopic areas; Joliot et al. 2015) in MRIcron. A summary of the location and size of participant lesions was obtained using the descriptive tool. Due to our small sample size, these data are underpowered for statistical analyses of lesion location and performance scores [e.g., voxel-based lesion-symptom mapping; (VLSM; Bates et al. 2003)]. Thus, while acknowledging the exploratory nature of the data, we include them for comparison with prior reports, and for the purpose of generating structure–function hypotheses (Danckert et al. 2012b; Stöttinger et al. 2014, 2015).

#### Statistical analysis

Data were analyzed using repeated measures ANOVA. In the online study, the mean percentages of first object reports (averaged over all sets per condition) were submitted to a repeated measures ANOVA with image number (15 morphing images from 100% first object to 0% first object) and condition (gradual vs. random) as within subject factors. Separate repeated measure analyses for each condition (gradual, random, repeat) were calculated for the patient study with the image number (15 morphing images from 100% first object to 0% first object) as a within subject factor and participant group (LBD, RBD and HCO) as a between subject factor. In Sect. Difference: random minus gradual, we calculate a difference score between first object reports in the random and gradual conditions (random–gradual) with positive numbers indicating a benefit for gradual presentations. The difference score was entered into a univariate ANOVA with participant group (LBD, RBD and HCO) as the independent variable. Analyses were calculated separately—(1) with the HCO-age-matched group, (2) with the HCO-cognitively matched group as controls and (3) restricted to RBD and LBD participant groups. Given that both patient groups suffered from brain injury this was considered the most meaningful comparison. Statistically significant main effects were further analyzed by a post-hoc Bonferroni tests as implemented in SPSS; *t* tests were used for post-hoc interaction analyses (Bonferroni corrected for multiple comparisons). Statistical test were two-tailed and an alpha level of *p* < .05 was used to determine significance.

In cases where the standard statistical tests failed to reject the null, we turned to Bayes Factors to assess whether the null was more probable than the alternative. Specifically, the Bayes factor allowed us to evaluate whether the differences between RBD and LBD represent (a) evidence for H1 [RBD performance being worse than LBD (Bayes factor ≥ 3)], (b) evidence for the null hypothesis of no performance difference (Bayes factor ≤ 0.33) or whether the data were not sensitive enough to confidently distinguish between the two alternatives (Bayes factor > 0.33 and < 3). Our cut-offs used were those recommended by Dienes (2014). One advantage of Bayes factors is their robustness to small sample sizes that are underpowered for conventional analyses (Vadillo et al. 2016). Bayes factors were calculated using the online-calculator provided by Dienes http://www.lifesci.sussex.ac.uk/home/Zoltan_Dienes/inference/Bayes.htm and where effect sizes came from our previous study (Stöttinger et al. 2014).

## Results

### Online study

**Fig. 3:**
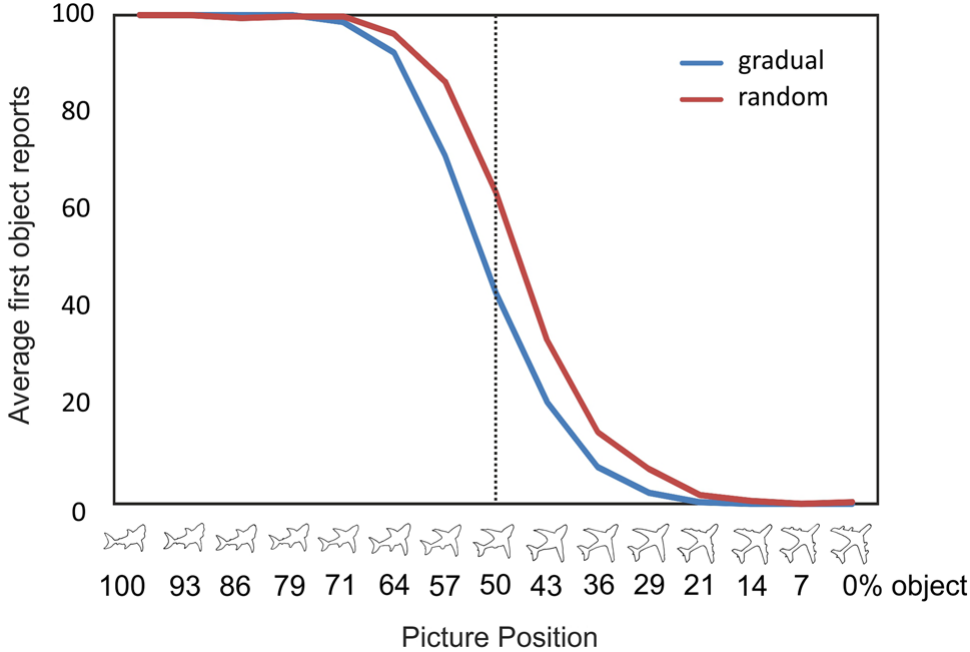
Average % of first object reports in the online study collapsed across all picture sets and all participants in each condition. The *x*-axis represents picture position (100% first object to 0% first object). Participants stopped reporting the first object earlier, when it was presented in the gradual condition (blue line) compared to the random condition (red line). At picture #8 (i.e., vertical dotted line), the picture was composed equally of both pictures (for interpretation of the references to color in this figure legend, the reader is referred to the web version of this article)

Seventy six of the 77 participants reported all catch trials correctly. Mean percentages of first object reports are displayed in Fig. 3. We refrained from comparing performance in the repeat condition with either the gradual or random conditions as morphing direction was not counterbalanced. A repeated measures ANOVA revealed a significant main effect for picture position [*F*(14,1064) = 2543.29, *p* < .001, *η*^2^ = .97]. As evident in Fig. 3, probability of first answer reports decreased as a function of picture position. A significant main effect for condition [*F*(1,76) = 31.35, *p* < .001, *η*^2^ = .29] showed that participants reported significantly fewer first objects in the gradual (mean 7.36, SD 0.76) compared to the random condition (mean 8.00, SD 0.72). The interaction between condition x picture position showed that participants reported the second object earlier in the gradual compared to the random context [*F*(14,1064) = 13.04, *p* < .001, *η*^2^ = .15]: at picture #7, 8, 9 and 11 probability of first object reports was significantly lower in the gradual compared to the random condition (all *p*’s < .05). These findings replicate Stöttinger et al. (2016).

### Patients

Patient participants identified all catch trials correctly, with the exception of one patient on one occasion. One set for one patient in the random condition was excluded from further analysis as the patient did not recognize one object (i.e., the patient failed to identify the spider in the spider–sun picture set even at the first picture position—100% spider/0% sun). See Table 3 for average performance in all three conditions for all groups (note: smaller numbers reflect better performance).

**Table 3:** Average performance of patients (a) and HCO (b)

ID	Gradual	Random	Repeat	Difference
(a) Performance of patients				
Left brain damaged patients				
110	6.00	8.50	7.50	2.50
442^c^	8.50	8.75	6.50	0.25
588	5.75	6.50	7.00	0.75
788	7.50	8.50	9.00	1.00
828	7.75	7.50	7.00	− 0.25
835	6.25	6.25	6.50	0.00
838	7.50	7.25	9.50	− 0.25
872	7.75	7.00	9.00	− 0.75
898	8.50	9.00	*10.00*	0.50
902	8.00	7.75	6.50	− 0.25
Right brain damaged patients				
27^a^	7.25	8.00	7.50	0.75
205^a^	8.50	7.75	8.00	− 0.75
228	*9.50*	7.00	8.50	− *2.50*
284^b^	*10.25*	6.75	7.00	− *3.50*
489^a^	8.50	*9.75*	8.00	1.25
729	7.50	7.25	6.00	− 0.25
744^b^	*9.25*	7.25	*10.50*	− *2.00*
792	*9.75*	6.75	*10.00*	− *3.00*
856	6.75	7.00	5.50	0.25
874	6.50	5.75	7.00	− 0.75
932	7.25	8.50	*11.50*	1.25
946	*9.25*	7.25	*10.00*	− *2.00*

ID	Gradual	Random	Random	Difference
(b) Performance of healthy controls				
HCO (≤ 70 years) (age-matched)				
1	6.25	7.25	6.00	1.00
46	5.75	7.00	5.50	1.25
110	6.75	7.25	9.50	0.50
143	6.75	7.50	7.00	0.75
193	6.25	7.50	5.50	1.25
230	6.25	8.00	7.50	1.75
249	6.25	8.50	6.00	2.25
351	6.00	7.75	7.00	1.75
408	7.50	7.25	6.00	− 0.25
HCO (>70 years) (cognitively-matched)				
3	5.75	8.00	8.50	2.25
32	6.25	7.25	6.00	1.00
37	7.75	6.80	7.50	− 0.95
148	7.25	7.75	8.50	0.50
206	7.50	8.25	8.50	0.75
208	8.00	7.00	5.50	− 1.00
321	5.00	6.75	6.00	1.75
369	6.75	9.50	8.00	2.75
409	7.00	7.25	8.50	0.25

^a^Neglect at time of screening (^b^and at time of testing): please note that there was no significant difference in any of the dependent measures between participants who showed neglect at screening (or at the time of this testing) and other RBD patients (all *p*’s > .05)

^c^Time since stroke was significantly longer than time since stroke for the other patients [*t*(20) = 15.58, *p* < .01]. This, however, did not result in better performance in this patient as evident in the table

Italic values represent performance 2 SDs outside the range of controls

#### Gradual condition

**Fig. 4:**
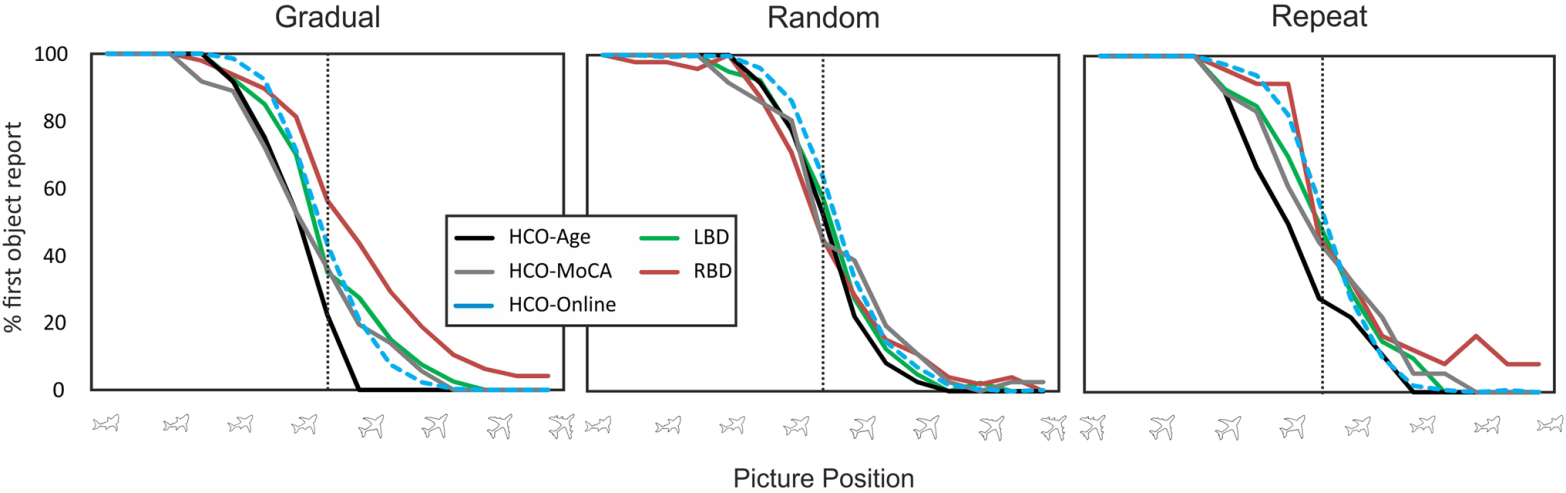
Average % of first object reports for gradual (left) random (middle) and repeat (right) conditions. The *x*-axis represents picture position (100% first object to 0% first object). At the vertical dotted line, the picture is composed of 50% first object and 50% second object. Green lines = LBD, red lines = RBD, black lines = HCO-Under 70, gray lines = HCO-Over 70. Results from the online study are included for comparison purposes (blue dotted lines = HCO-online) (for interpretation of the references to color in this figure legend, the reader is referred to the web version of this article)

Compared to the HCO-age-matched group, results demonstrated a significant main effect for image number [*F*(14,392) = 353.04, *p* < .001, *η*^2^ = .93] due to a decrease of first object reports as a function of picture position, as well as a significant main effect for participant group [*F*(2,28) = 9.47, *p* = .001, *η*^2^ = .40] (Fig. 4, left panel). A post hoc Bonferroni test showed that age-matched HCO and LBD patients had a comparable percentage of first object reports (*p* = .16). Age-matched HCO had a significantly lower percentage of first object reports than RBD patients (*p* = .001). This analysis also demonstrated a significant interaction between image number and participant group [*F*(14,392) = 3.53, *p* < .001, *η*^2^ = .20]. Independent samples *t* test (Bonferroni corrected) revealed that both patient groups showed a significantly higher percentage of first object reports at picture image #9 and 10 compared to HCO-age matched controls.

The same repeated measures analysis restricted to patient groups (i.e., RBD vs. LBD) demonstrated a significant main effect for picture position [*F*(14,280) = 206.97, *p* < .001, *η*^2^ = .91] and a marginally significant main effect for participant group: RBD patients tended to have a higher proportion of first object reports compared to LBD patients [*F*(1,20) = 4.12, *p* = .056, *η*^2^ = .17]. To further evaluate the difference in LBD and RBD first object reports, we calculated the Bayes factor (Dienes 2014). We found a Bayes factor of 3.24, indicating support for a greater updating impairment after damage to the right side of the brain.

The same analysis conducted with the HCO-cognitively matched group revealed similar results. There was a significant main effect for image number [*F*(14,392) = 301.09, *p* < .001, *η*^2^ = .92] and participant group [*F*(2,28) = 5.33, *p* = .011, *η*^2^ = .28]. Age-matched HCO had a comparable percentage of first object reports to LBD patients (*p* = .88), and a significantly lower percentage of first object reports than RBD patients (*p* = .01).

This analysis also showed a significant interaction between picture position and participant group [*F*(28,392) = 1.55, *p* = .038, *η*^2^ = .10]. HCO-cognitively matched controls showed a comparable percentage of first object reports to LBD patients at all picture positions (all *p*’s > .05), and significantly lower percentage of first object reports than RBD patients at picture #7 (*p* = .048) (Fig. 4).

#### Random condition

The same analyses conducted for averaged percentage of first object reports in the random condition revealed a significant main effect for image number for both analyses—with age-matched HCO as controls [*F*(14,392) = 403.85, *p* < .001, *η*^2^ = .94] and with HCO matched on cognitive impairment [*F*(14,392) = 361.12, *p* < .001, *η*^2^ = .93]. Percentage of first object reports decreased as a function of picture position in both analyses. No other effects or interactions were significant (all *p*’s > .05) indicating that all groups performed at equivalent levels (Fig. 4). A Bayes factor of 0.13 for the difference between RBD and LBD patients confirmed the likelihood of equal performance between the two patient groups.

#### Repeat condition

The percentage of first object reports in the repeat condition was submitted to the same repeated measures ANOVA described above and revealed a significant main effect for image number with HCO-age-matched as controls [*F*(14,392) = 140.03, *p* < .001, *η*^2^ = .83]: the percentage of first object reports decreased as a function of picture position. We also found a trend towards a significant effect for participant group [*F*(2,28) = 2.82, *p* = .077, *η*^2^ = .17]. HCO-age-matched controls showed a slight, but not significantly lower percentage of first object reports compared to RBD patients (*p* = .075), and a comparable performance to LBD patients (*p* = .76). Calculating the analysis for patient groups only (RBD vs. LBD) revealed a significant main effect for picture position, again showing a decrease of first object reports as a function of picture position [*F*(14, 280) = 99.94, *p* < .001, *η*^2^ = .83]. No other main effect or interaction reached significance (all *p*’s > .05). While RBD and LBD patients demonstrated comparable performance, a Bayes factor of 0.57 suggest that our data is not sensitive enough to draw a definite conclusion of no patient group differences.

The same repeated measures ANOVA conducted with HCO-cognitively-matched controls revealed no significant effect other than a significant main effect for image position [*F*(14,392) = 141.84, *p* < .001, *η*^2^ = .84]. No significant difference was found for average percentage of first object reports between HCO-cognitively matched and LBD (*p* = 1) or RBD patients (*p* = .68). Results therefore demonstrate that when the control group was matched for cognitive impairment, no difference was found between groups (Fig. 4).

#### Difference: random minus gradual

**Fig. 5:**
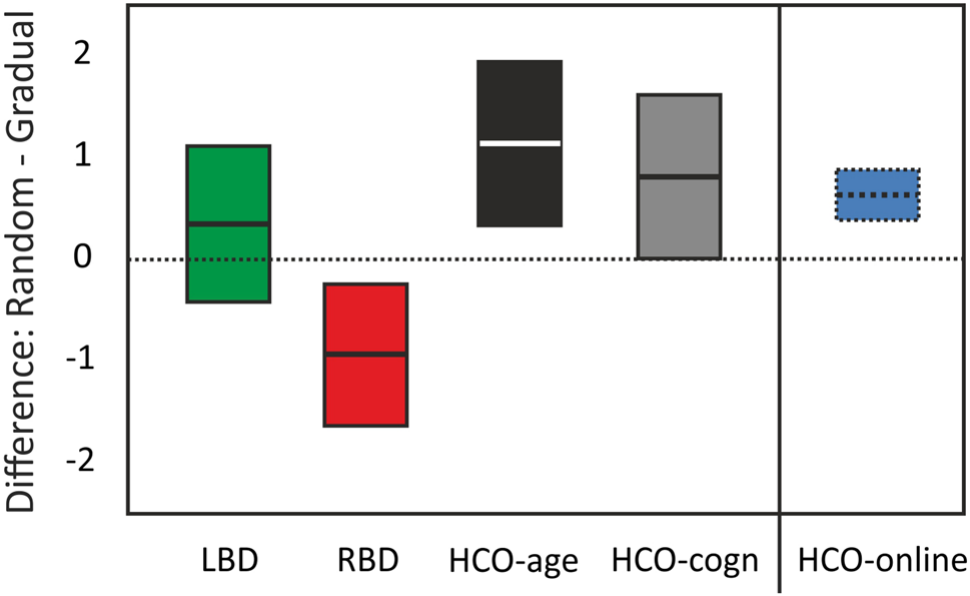
Average (95% CI) difference scores (random–gradual) for LBD (green), RBD (red), HCO-age-matched and HCO-cognitively-matched (gray). Results of healthy young participants from the online study are included for comparison purposes (blue) (for interpretation of the references to color in this figure legend, the reader is referred to the web version of this article)

Healthy individuals see the second object earlier when the pictures are presented in a gradual morphing context (Fig. 3). We submitted the difference scores (average proportion of first object reports in random–gradual conditions; Table 3) to two separate univariate ANOVAs with participant group (RBD, LBD, and either HCO-age matched or HCO-cognitively matched) as the between subject factor. Larger values for this difference score indicate greater benefit from the gradual condition. The analysis with HCO-age matched as a control group showed a significant main effect (Fig. 5) [*F*(2,28) = 7.74, *p* = .002, *η*^2^ = .36]. RBD patients had significantly smaller difference scores compared to LBD patients [*F*(1,20) = 4.82, *p* = .04, *η*^2^ = .19] and HCO-age-matched controls: *F*(1,19) = 12.25, *p* = .002, *η*^2^ = .39; LBD patients tended to have smaller difference compared to age-matched HCO [*F*(1,17) = 4.12, *p* = .058, *η*^2^ = .20].

The same analysis done for HCO-cognitively-matched group as controls also showed a significant main effect [*F*(2,28) = 4.85, *p* = .016, *η*^2^ = .26]. HCO-cognitively matched controls showed a significantly larger benefit for gradual vs. random presentation than did RBD patients [*F*(1,19) = 6.88, *p* = .017, *η*^2^ = .27] and a comparable benefit to LBD patients [*F*(1,17) = 0.81, *p* = .381, *η*^2^ = .05].

#### Healthy controls—age effects

The two HCO groups differed on age in the gradual condition only. The HCO-age-matched (≤ 70 years) group stopped reporting the first object earlier compared to the HCO-cognitively-matched (> 70 years) counterparts as evident in a significant interaction between image number x participant group (age matched vs. cognitively matched) [*F*(14,224) = 1.92, *p* = .026, *η*^2^ = .11] (Fig. 4, left panel). None of the other conditions revealed a significant difference between the two control groups (all *p*’s > .05).

#### Lesion overlay analysis

**Fig. 6:**
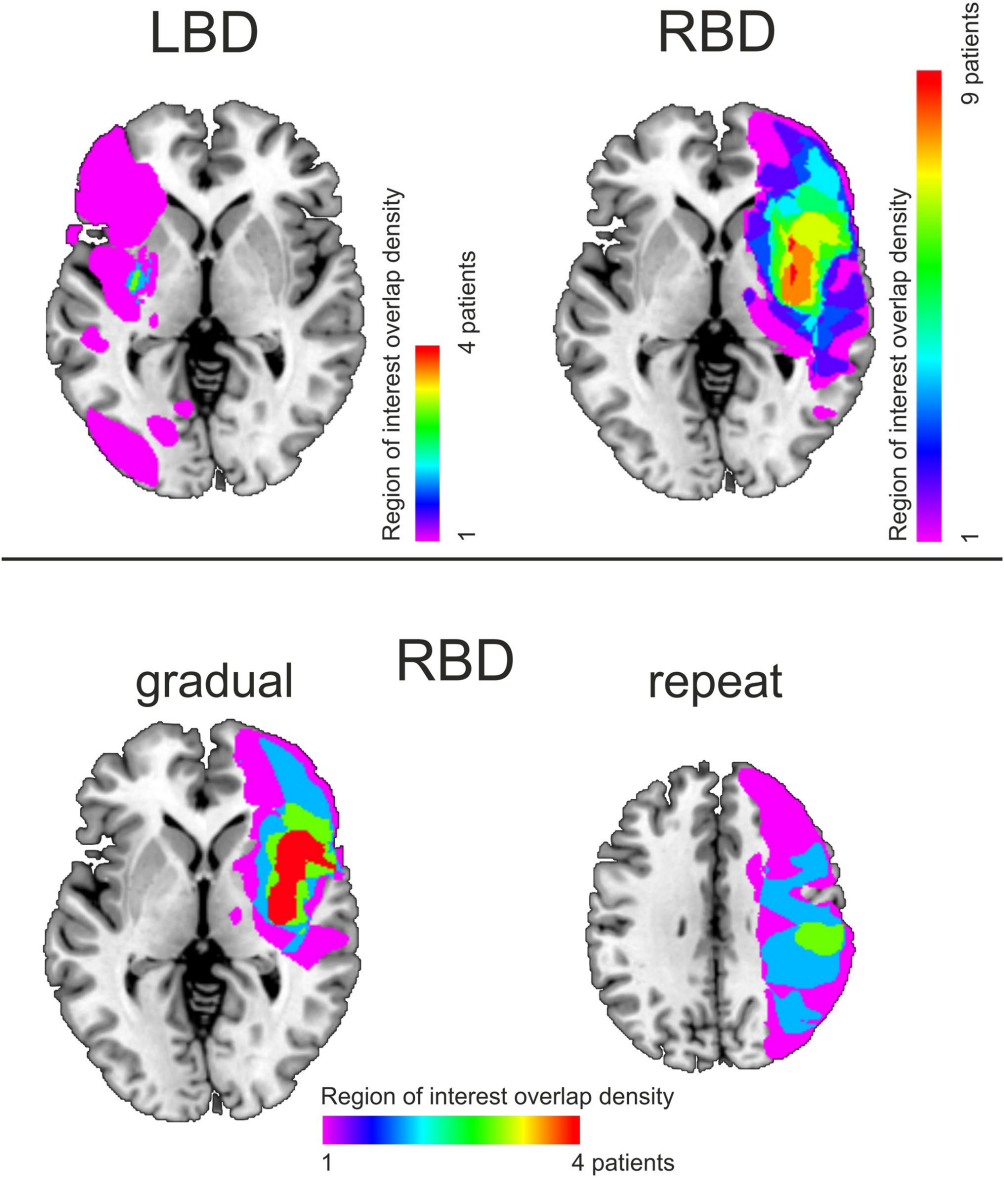
Lesion overlay maps for LBD and RBD patients (top panel). Bottom panel shows 4 of the 5 RBD patients who were considered poor explorers (left panel) and 3 of the 4 RBD patients, considered poor proactive updaters (right panel). Shading indicates the amount of overlap

Among the 12 RBD patients, nine had overlapping lesions in the insula (anterior and posterior), rolandic operculum, and the putamen. Five RBD patients were considered poor explorers based on average first object reports and difference scores more than two SDs above the mean of the HCO-cognitively matched group (highlighted by gray bars in Table 3). Four of these five patients had common involvement in the inferior frontal cortex, precentral gyrus, insula (anterior and posterior), rolandic operculum, superior temporal gyrus, and the putamen (Fig. 6). Four RBD patients showed a poor general switching performance as indicated by average first object reports that were two SDs above the mean of the HCO-cognitively matched group in the repeat condition. Three of these patients had overlap in the parietal cortex (postcentral, supramarginal gyrus, angular gyrus, inferior parietal gyrus, intraparietal sulcus), and the posterior insula and superior temporal gyrus (Fig. 6). LBD patients demonstrated less overlap in their lesions than did the RBD group with only four of ten patients showing common involvement of the putamen.

## Discussion

The aim of the present work was to explore *why* RBD patients show a selective and differential impairment of mental model updating, and to replicate results of our earlier study (Stöttinger et al. 2014). As in our previous study, RBD patients needed significantly more pictures than HCO before they reported seeing the second object in the gradual morphing condition—even after controlling for general cognitive impairment (Fig. 4). Comparisons between LBD and RBD groups found clear evidence for worse updating in RBD patients (Bayes Factor 3.24). Further, five of the 12 RBD patients, but none of the ten LBD patients, performed well outside the average performance of the control group (i.e., mean of HCO-cognitively-matched ± 2 SD; Table 3).

To test whether the updating impairment in RBD patients reflects (1) an inability to explore new hypotheses, (2) a general impairment of proactive switching or (3) a more general perceptual and/or attentional impairment, we compared three conditions: gradual, random and repeat. All groups performed at the same level in the random condition, indicating that basic perceptual, attentional, and language capacities were equivalent in all participant groups (Bayes factor of 0.13). To test whether RBD performance was due to impaired ability to explore alternative interpretations, we compared first object reports in the random condition with the gradual condition. In healthy individuals, we consistently find that participants report the second object earlier when presented in a gradual context (Stöttinger et al. 2016; Fig. 3), reflective of active exploration strategies (i.e., “It was a shark but it could also be a plane or a bird.”). In the online study, we replicated this benefit showing the robustness of this effect. This is an important point given the ongoing discussion about failures to replicate in social science (Bohannon 2015). Results in the patient study showed a significant difference between participant groups in how much they benefited from the gradual presentation. The strongest effect was seen in healthy age-matched controls. This benefit decreased with age and was about the same in the older seniors (matched on general cognitive impairment) as in LBD patients (Fig. 5). In contrast, RBD patients exhibited a perceptual hysteresis—becoming “stuck” on their initial interpretation even as the pictures gradually morphed into something else. Combined with our previous work (Danckert et al. 2012b; Stöttinger et al. 2014), this indicates that RBD participants are aware that things are changing in the perceptual representation they are forming, but are deficient in exploring alternative models to encapsulate those changes. That is, our RBD patients’ verbal reports of the morphing images indicate that they perceive changes. What differentiated them from controls and LBD patients was that they interpreted those changes within the context of their initial perceptual representation. Indeed, if the task we employed depended heavily on detecting the small (~ 4%) changes from one image to the next, one might assume that it would be LBD that would lead to the greatest impairment, as damage to this hemisphere is known to affect local as opposed to global image processing (Martinez et al. 1997; Robertson and Lamb 1991). Therefore, we suggest that the deficit seen here in RBD patients is indicative of a failure to adapt to changing circumstances as they struggle to explore alternate hypotheses to explain the change.

Data in the repeat condition are harder to interpret given the smaller number of observations (i.e., two instead of four sets). In the repeat condition, there is no need to explore alternate hypotheses—participants know that the plane will morph into a shark; they have seen it before. All that remains is for participants to determine when to report seeing the shark. Performance of RBD patients as a group was not different from that of LBD patients or cognitively matched controls in the repeat condition (Fig. 4). However, our rejection of the proactive switch account rests on the strength of a null result, and our Bayes factor of 0.57 suggests that these negative data are not strong. Further comparisons of gradual and repeat conditions will be necessary to evaluate whether updating impairments in RBD patients are due only to failures of hypothesis exploration, proactive switching, or both.

Although RBD patients performed more poorly than HCO and LBD patients, it is also evident that performance within the RBD patients was heterogeneous (Table 3). Some RBD patients performed within the norm of cognitively-matched controls, while others were outside this range. There was also heterogeneity in the type of impairment observed, with some patients only impaired in one regard (e.g., exploring) while others had broader impairments (Table 3). Given our sample sizes the lesion overlay analyses should be regarded as exploratory. Nevertheless, they are consistent with general ideas about the systems important for adapting to changing and uncertain circumstances. A failure to explore was mostly associated with damage to frontal regions (frontal operculum, insula), while a general impairment in alternating between interpretations (as indexed by the repeat condition) was associated more prominently with parietal damage (Fig. 6). In healthy individuals, an association between the right insula—commonly damaged in our patients—and exploration of alternative interpretations in stochastic and uncertain environments has been found by several groups (Blanchard and Gershman 2018; Ohira et al. 2013, 2014; Laureiro-Martínez et al. 2015). When we presented healthy individuals with some of the gradually morphing picture sets used here in an fMRI experiment, we found that the anterior insula was active not only at the actual time point of reported object change but also about five seconds before—consistent with the involvement of the anterior insula in the exploration of alternative choices (Stöttinger et al. 2015). We have recently replicated this finding (Stöttinger et al. 2018).

By directly comparing HCO groups, we found that the ability to recognize the second object is sensitive to age, with older seniors needing significantly longer than younger seniors to identify the second object in the gradual morphing condition. This is consistent with findings showing slower switch rates in ambiguous figures as people age (Ukai et al. 2003). Since these groups were selected to match either the age or the cognitive status of the patient groups, it cannot be conclusively stated that age affects updating behavior. This effect could also reflect general cognitive decline. Indeed, six of our 18 HCO had a MoCA score below 26 with four of these participants in the HCO-cognitively matched group (> 70 years). According to the cutoff point initially published, this would be suggestive of a mild cognitive impairment (MCI; Nasreddine et al. 2005; Smith et al. 2007). However, more recent studies suggest cut-off scores of 22/23 to be more sensitive for MCI (Lee et al. 2008; Luis et al. 2009). All of our HCO were well within this range.

In summary, our study suggests that updating impairments seen after damage to the right side of the brain most likely represent an impairment in the ability to explore the alternative interpretations within a slowly changing environment. A better understanding of this type of cognitive impairment is relevant for developing an improved understanding of post-stroke behavioral deficits and prompting the study of specific rehabilitation procedures.
